# Demonstration of a terahertz coplanar-strip spoof-surface-plasmon-polariton low-pass filter

**DOI:** 10.1038/s41598-023-50599-y

**Published:** 2024-01-02

**Authors:** Mohsen Haghighat, Thomas Darcie, Levi Smith

**Affiliations:** 1 Department of Electrical and Computer Engineering, University of Victoria, Victoria, BC V8P 5C2 Canada; 2 Centre for Advanced Materials and Related Technology (CAMTEC) at the University of Victoria, 3800 Finnerty Rd, Victoria, BC V8P 5C2 Canada

**Keywords:** Microwave photonics, Metamaterials

## Abstract

There is a growing interest in spoof surface plasmon polariton (SSPP) structures at terahertz (THz) frequencies for applications such as filtering, sensing, and communications. However, to date, there are limited experiments that confirm SSPP characteristics at THz frequencies. The majority of literature focuses on simulation or verification by device scaling to Gigahertz (GHz) frequencies where standard vector network analyzers are readily available. This paper presents the first experimental verification of SSPP characteristics at THz frequencies in a guided wave system using coplanar strip (CPS) feedlines. Specifically, we design three SSPP structures with varying band-edge frequencies (1.04 THz, 0.63 THz, and 0.53 THz), then fabricate and verify the low-pass transmission characteristics using a modified THz-time-domain spectrometer (THz-TDS) system. We find strong agreement between simulation, theory, and experiment.

## Introduction

Terahertz (THz) systems and devices have garnered extensive research attention over the past few decades due to their attractive applications in sensing, imaging, data communication transceivers, and biomedicine^1–3^. This research has also sparked significant interest in spoof surface plasmon polaritons (SSPP) structures which mimic the behavior of conventional surface plasmon polaritons (SPP)^4,5^. SSPPs differ from conventional SPPs in that their dispersion characteristics are primarily determined by geometry as opposed to material parameters which enable SSPPs to exist at sub-optical frequencies. We note that both SSPPs and SPPs are capable of supporting surface-wave propagation and sub-wavelength field localization. SSPPs can be used for several applications, but the standard use cases are filtering, sensing, and communications^3^. Regarding filtering, SSPP devices exhibit low-pass behavior with significant roll-off rates over short structure lengths. Practically, short filter lengths are desirable to minimize the necessary component area. For sensing, SSPPs exhibit slow-wave behavior and significant field enhancement near the component^6^. The combination of these effects in the presence of an unknown sample can be used for material parameter identification^7^. Lastly, for communications, SSPPs have a relatively low loss (0.75 dB/mm at 0.22–0.32 THz^8^) and are resilient to bend losses^9^. This is an important advantage because bend losses limit the practicality of other single conductor waveguides such as the Goubau line^10^ or single wire waveguide^11^.

It is not straightforward to directly excite the fundamental mode transverse magnetic (TM) of an SSPP waveguide. For practical purposes, SSPP structures are typically integrated with a feedline such as a coplanar waveguide (CPW)^12–14^, coplanar strip (CPS)^15–17^, microstrip (MS)^18^, or slotline (SL)^19^. The fundamental modes of the aforementioned waveguides are quasi-transverse electromagnetic (TEM) (CPW, CPS, and MS) or quasi-transverse electric (TE) for the SL^20^. Given that the feedline and SSPP have different modes, a transition circuit (TC) is necessary to perform a mode conversion. To date, most of the research efforts have utilized a CPW feedline to excite an SSPP mode where large flaring grounds are used in the TC to aid in the mode conversion^20^. The CPW configuration is commonly used because of its compatibility with standard microwave frequency vector network analyzers (VNA) probes. Alternatively, in^15^, it was suggested to use a CPS feedline to excite the SSPP mode (termed CPS-SSPP). The CPS-SSPP has several benefits such as a reduced circuit size and improved lumped element compatibility. In^15^, the authors designed a *THz* CPS-SSPP structure but fabricated and tested a scaled structure at *GHz* frequencies. The reason for the scale modification is that it is exceedingly difficult to perform device characterization at THz frequencies because there is limited or no commercial equipment available. Downshifting to GHz provides useful insight, but it negates difficulties that are expected to occur at THz frequencies such as substrate radiation and extraneous resonances. Also, if the proposed application of an SSPP is a chemical sensor, then it is of the utmost importance that the device operates where the molecular absorption signatures reside, for vibrational resonances it is typically at THz frequencies^21^.

While many simulations at THz frequencies have been conducted, most of the validation experiments have been performed at GHz frequencies by scaling the structures and implementing on MS platforms to measure the performance up to 16 GHz^15–17,22^. To date, experimental measurements of THz SSPPs have predominantly focused on frequencies up to approximately 300 GHz^8,12,23^ except for our preliminary work which focused on a single CPS-SSPP configuration^24^. On the other hand, some studies have employed radiated wave approaches to investigate SSPPs at frequencies greater than 1 THz^25^; however, this work focuses on using non-radiative feedlines for their improved integration benefits such as their well-known field profiles and planar coupling efficiencies.

**Figure 1:**
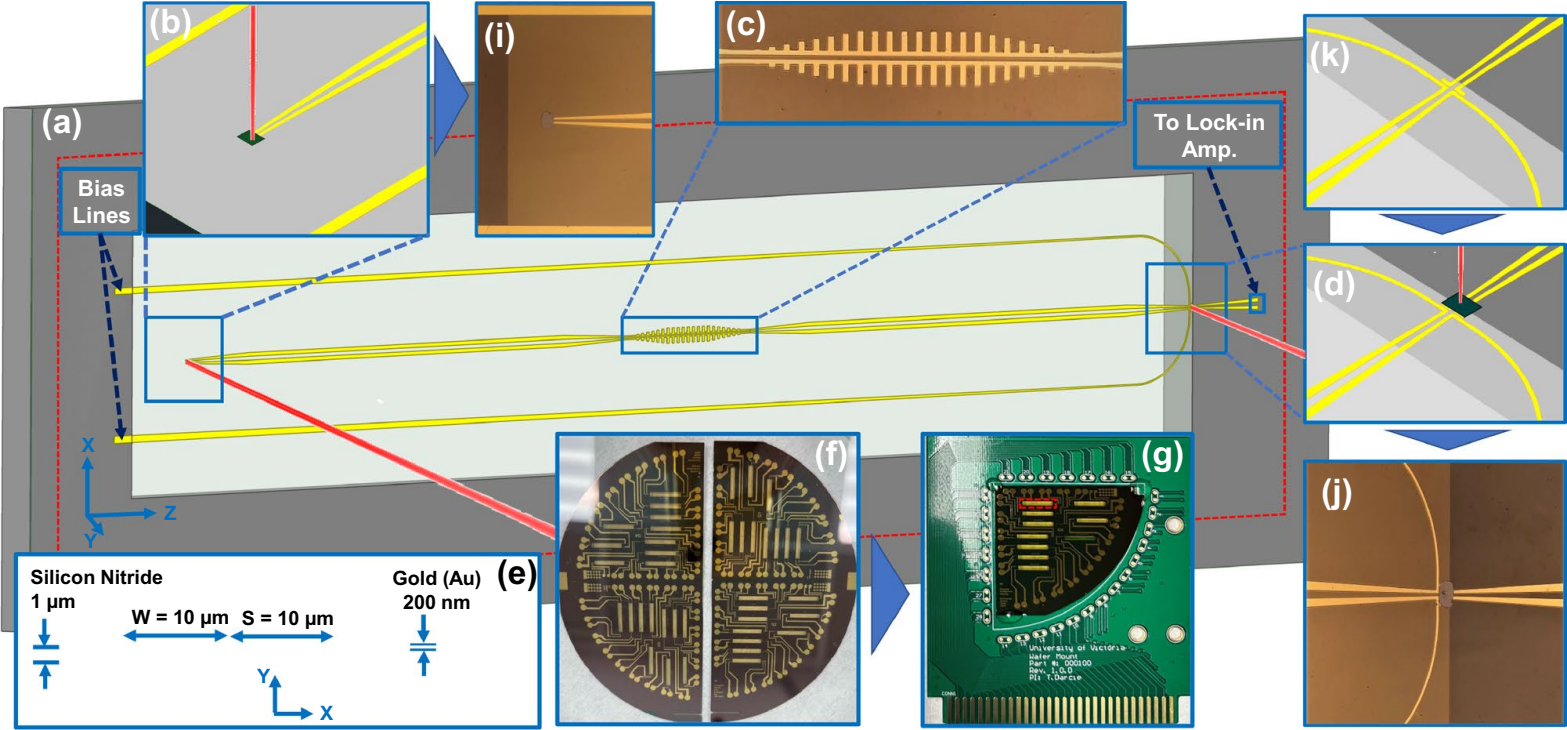
Illustration of CPS-SSPP device and measurement platform. (**a**) The whole structure along with tapered and bias lines on Silicon Nitride membrane. (**b**) Transmitter PCS and incident laser beam. (**c**) Main CPS-SSPP structure. (**d**) Receiver PCS and incident laser beam. (**e**) Dimensions of central reference CPS line and the membrane. (**f**) Fabricated circuits on Wafer. (**g**) Mounted Wafer quarter on PCB to connect to the measurement setup. (**i**) Real transmitter PCS. (**j**) Real receiver PCS. (**k**) Contact point for the receiver PCS on the edge of the membrane.

To verify SSPP behavior, we investigate a signal that has been transmitted through an SSPP. It is known that an SSPP behaves like a low-pass filter where the cut-off frequency is given by the band-edge associated with the periodic structure (discussed later)^5^. In this work, we integrate three different CPS-SSPP geometric configurations into our THz System-on-Chip (TSoC) platform^26^ as shown in Fig. 1, then we verify the stop-band location at THz frequencies. The TSoC platform consists of planar conductors on a very thin (1 µm) Si$_3$N$_4$ membrane which reduces the loss and dispersion of the propagating wave along the CPS feedlines and ensures the CPS-SSPP is excited with a near-TEM field profile. This method enables us to characterize devices at THz frequencies with ease since radiative substrate losses are heavily mitigated. We find that the SSPP results from the experiments are in agreement with simulation and theory. To the best of our knowledge, this work presents the highest experimental frequency achieved for guided-wave SSPP characterization. Note that this is a proof-of-concept, and with the appropriate circuit modifications, it is possible to investigate SSPP device characteristics beyond 3 THz. The most salient feature of the presented TSoC CPS-SSPP is the ease of implementation while obtaining THz-band operation. While it is possible to use a VNA along with several extension modules as an alternative, it becomes a costly endeavor which currently cannot exceed 1.5 THz using commercially available extension modules.

## Results

## Theory of the CPS-SSPP and dispersion characteristics

**Figure 2:**
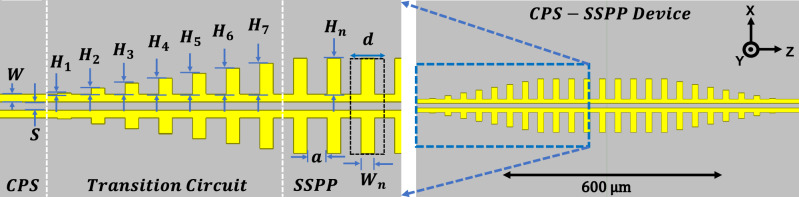
Transition circuit (TC) connected to the CPS-SSPP structure. $W_n$ = 20 µm, *a* = 30 µm, *W* = 10 µm, and *S* = 10 µm. See Table 1 for stub height dimensions.

The analysis and modeling of several SSPP structures have been investigated in prior work^27^. Unfortunately, to our knowledge, no closed-form dispersion relations have been presented for a finite-thickness CPS-SSPP structure. However, if the thickness of the CPS-SSPP conductors were to become infinite, then the CPS-SSPP dispersion relation can be approximated by a 1D array of grooves^5,9^:

1 $$\begin{aligned} k_z= k_{eff} \sqrt{1 + \left( \frac{a^2}{d^2}\right) {\text {tan}}^2(k_{eff}H_n)}, \end{aligned}$$

where $H_n$ is the length of stubs (i.e. depth of grooves) in the SSPP region (see Fig. 2), $d = a + W_n$ is the period, *a* is the aperture or the distance between the stubs, $W_n$ is the width of the stubs, $k_{eff} =\omega \sqrt{\varepsilon _{eff}}/c$ is the effective wavenumber, *c* is the speed of light, and $\varepsilon _{eff}$ is approximated by the effective relative permittivity of the CPS feedlines at THz frequencies. There are empirical models to obtain $\varepsilon _{eff}$^28–30^, but we use numerical simulations to overcome the constraints of the empirical boundaries. For the CPS TLs in this work, we find $\varepsilon _{eff} \approx 1.7$ between 0.1 and 1.5 THz using ANSYS HFSS. We note that (1) is not explicitly true for a CPS-SSPP, but it provides a reasonable approximation. The validity of a similar approximation is also found in^9,13^.

Investigation of (1) reveals that the CPS-SSPP exhibits low-pass behavior (when $k_{eff} H_n$ is small, then $k_{z} \approx k_{eff}$). Next, we see that the response of SSPP structures is primarily controlled by $H_n$. This is observed by considering $k_{eff} H_n \rightarrow \pi /2$ which results in $k_z \rightarrow \infty$. We note that this relates to the asymptotic frequency which is calculated by $f_{AS}=c/(4\sqrt{\varepsilon _{eff}}H_n)$. At frequencies below the band-edge ($k_{z} d = \pi$), the insertion loss will be minimal. At frequencies above the band edge, a significant insertion loss is expected. Near the band-edge, a resonance occurs which results in large field enhancement inside the CPS-SSPP grooves^15^.

### Design of the CPS-SSPP structure

This paper complements the CPS-SSPP proposed in^15^ and thus our design was influenced by their work. Our primary goal is to demonstrate low-pass behavior for several different CPS-SSPP configurations at THz frequencies. Fortunately, the theory presented in the previous section provides a reasonable estimate of the expected band-edge frequency. As noted, $H_n$ is the main parameter that controls the band-edge frequency. In this work, we fabricate three different CPS-SSPP structures where $H_n$ = 45 $\upmu$m, 85 $\upmu$m, and 105 $\upmu$m. Inserting these values (1), then numerically solving predicts band-edge frequencies to be 1.04 THz ($H_n$ = 45 $\upmu$m), 0.63 THz ($H_n$ = 85 $\upmu$m), and 0.53 THz ($H_n$ = 105 $\upmu$m). Here, we specified the geometric parameters, and then calculated the frequency response, however, the converse could also have been performed to obtain a desired band-edge frequency. Next, the period of corrugation, *d*, and aperture, *a*, should be subwavelength^5,6^. To retain a level of comparability amongst other SSPP literature, we selected to use *d* = 50 µm and $a = 0.6d = 30$ µm^5,6^. The designer can select $0.1 \le a/d \le 0.9$ depending on the desired dispersion characteristics given by (1). For filtering applications where a sharp roll-off is desired, a larger value of *a*/*d* is preferred since the dispersion curve approaches the asymptotic frequency more rapidly, however, this will impact the phase response and pulse shape. Also recognize that increasing *a*/*d* results in narrow stubs which can result in poor stop-band rejection. Note that *a*/*d* does not affect the asymptotic frequency and it has negligible impact on the band-edge frequency based on (1).

The transition circuit (TC) shown in Fig. 2 is required to provide the gradient momentum matching between the CPS feedlines quasi-TEM mode and the SSPP TM mode^13,15^. This concept can be described using (1) and observing two cases: $H_n = 0$ and $H_n \ne 0$. When $H_n = 0$ the CPS-SSPP is equivalent to a typical CPS TL and $k_z/k_{eff}=1$ suggesting the momentum is matched which results in no reflections. Alternatively, when $H_n \ne 0$, then $k_z/k_{eff} > 1$ which implies a discontinuity in momentum which results in mode conversion. The gradual introduction of the CPS-SSPP stubs (i.e., the TC) reduces the discontinuity and improves the mode conversion efficiency. Supplementary Material (S1) illustrates the impact of negating the TC on the transmission. The CPS feedline dimensions were selected to be *S* = *W* = 10 µm to ensure the SSPP dimensions could remain on a sub-wavelength scale. Table 1 tabulates the dimensions for the TC and CPS-SSPP. The number of SSPP stubs is *N* = 9, although it can be higher with no significant impact on the frequency response^15^.

This work performs the verification of SSPP behavior using our TSoC platform. Regarding the CPS-SSPP, key design considerations are TSoC material and geometric parameters. The TSoC uses 1 µm thick Si$_3$N$_4$ membrane substrate to minimize loss and dispersion of the feedlines required to drive the CPS-SSPP^26^. The conductors are 210 nm (10 nm Ti, 200 nm Au) and are defined by photolithography. The transmitter and receiver are thin-film photo-conductive switches (PCSs) made from LT-GaAs. The average optical power on the transmitter is 7 mW, and the average optical power on the receiver is 14 mW. More details are provided in the Fabrication section.

**Table 1:** Stub lengths for the TC (units: µm).

$H_n$	$H_1$	$H_2$	$H_3$	$H_4$	$H_5$	$H_6$	$H_7$
45	1	8	14	20	26	33	39
85	6	18	29	40	51	63	74
105	9	23	36	50	64	78	91

### Eigenmode and frequency domain simulations

To complement the theory and experiment we use ANSYS HFSS to perform eigenmode and frequency-domain (FD) full-wave simulations of the CPS-SSPP structures. In all simulations we aim to replicate the real devices, thus we model the Si$_3$N$_4$ substrate as 1 µm thick and $\varepsilon _r$ = 7.6, $\sigma _{Si_3N_4}=0$, and tan $\delta _e$ = 0.00526^31^. The gold thickness is 200 nm and the conductivity is $\sigma _{Au} = 4.1 \times 10^7$ S/m. All simulations use $d =$ 50 µm, $W_n$ = 20 µm, *S* = 10 µm, and *W* = 10 µm. The stub dimensions are found in Table 1.

**Figure 3:**
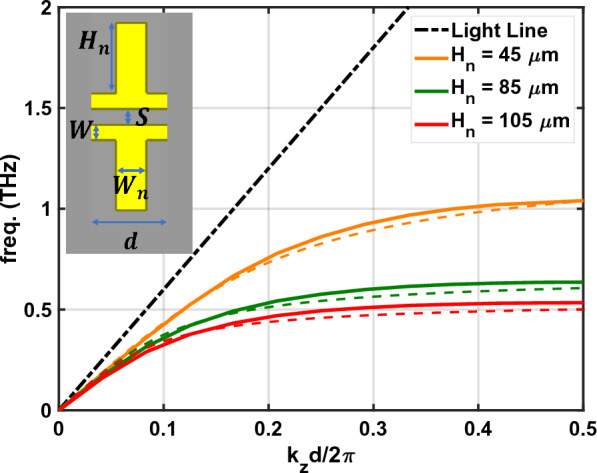
Dispersion curves obtained by eigenmode simulation (solid lines) and theory (dashed lines) on the CPS-SSPP unit cell (inset). $H_n$ is variable as shown in the legend and fixed dimensions are $d =$ 50 µm, $W_n$ = 20 µm, and $S =$ 10 µm.

We use the eigenmode simulation to obtain the dispersion diagram and band-edge frequencies for the CPS-SSPP unit cells. Figure 3 plots the simulated dispersion relation (solid lines) for the three CPS-SSPP structures investigated in this work. It is possible to use (1) for this purpose (dashed lines), but we reiterate that (1) was derived for a 1D array of grooves, thus caution is required. Regardless, for each case, we see asymptotic behavior which is characteristic of SSPPs. The simulated band-edges were found to be: 1.04 THz ($H_n$ = 45 µm), 0.63 THz ($H_n$ = 85 µm), and 0.53 THz ($H_n$ = 105 µm) which are in close agreement with values previously obtained from (1).

**Figure 4:**
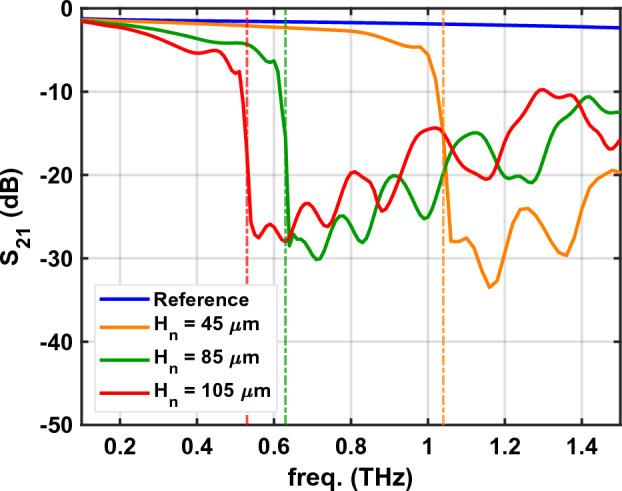
CPS-SSPP $S_{21}$ (dB) from FEM simulation results for the structures which were experimentally tested. The vertical dashed lines indicate the simulated band-edge frequencies.

**Figure 5:**
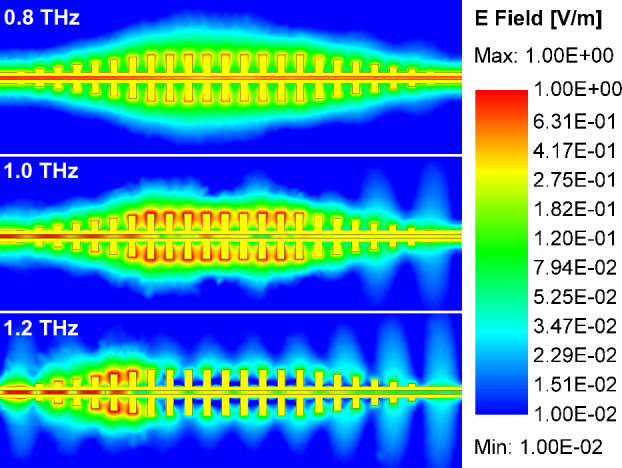
Field plots of CPS-SSPP at different frequencies on the structure with $H_n$ = 45 µm. (**a**) 0.8 THz, on pass band. (**b**) 1.0 THz on the pass band, below the band-edge frequency, with maximized field confinement in the grooves. (**c**) 1.2 THz, on stop band. (The color scale is logarithmic and normalized between 0.01 and 1.00).

We perform FD simulations for several purposes. First, we investigate the transmission through the fabricated CPS-SSPP structures where $H_n$ = 45, 85, and 105 µm. Figure 4 plots the results of these simulations. Low-pass behavior is clearly observed and the cut-off frequency aligns with the predicted band-edge frequencies. Next, we use FD simulations for illustrative purposes. Figure 5 shows the electric field at frequencies below cut-off (0.8 THz), near cut-off (1.0 THz), and above cut-off (1.2 THz). As expected, near cut-off, a large localized field is observed in the SSPP grooves. Lastly, Supplementary Material (S2) presents the impact of using another substrate for literary comparison with^15^ and demonstrates the impact on the band-edge frequency.

### Measurement results of the CPS-SSPP

**Figure 6:**
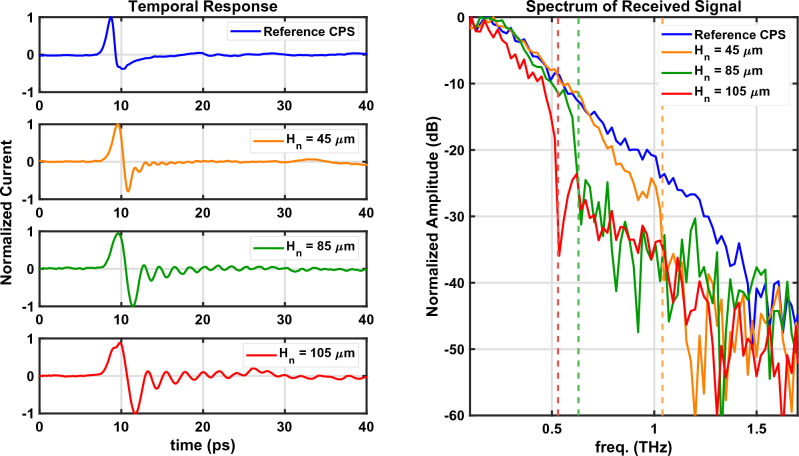
Measurement results for structures with different stub lengths. The time-domain results are obtained from the lock-in amplifier, the spectral response is obtained by applying the Discrete Fourier Transform of the temporal response. The vertical dashed lines indicate the simulated band-edge frequencies.

**Figure 7:**
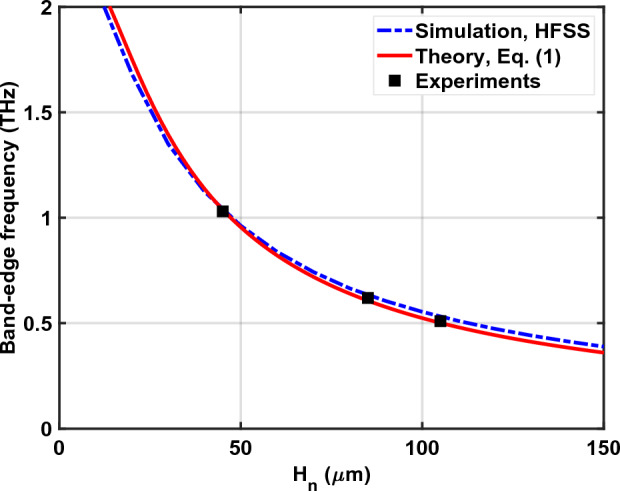
Band-edge frequency versus $H_n$ obtained from eigenmode simulations, theoretical dispersion relation, and experiment.

The experimental results for the CPS-SSPP are displayed in Fig. 6 alongside a reference without stubs (i.e., $H_n=0$). We note a discrepancy between the experimental results (Fig. 6) and simulated results (Fig. 4) which originates from different incident signals. For the experiment, the incident signal is a sub-picosecond time-domain pulse which has an inherent roll-off whose spectral response resembles the Reference signal in Fig. 6. To estimate the experimental signal transmission, it is necessary to take the difference between the respective signal ($H_n$ = 45, 85, or 105 µm) and the common reference measurement. Alternatively, for the frequency-domain simulation (Fig. 4), the incident signal is frequency-independent and well-known thus the simulation directly outputs the transmission. The vertical dashed lines in Fig. 6 indicate the simulated band-edge frequencies which align the transmission cut-off as predicted in Fig. 4. We see that as the stub length increases the cut-off frequency reduces as predicted by (1). Figure 7 plots the band-edge frequency versus $H_n$ using simulation, theory, and experiment where reasonable agreement is observed. Next, the roll-off rate associated with the band-edge frequencies is significant and is on the order of $\approx$ -160 dB/Octave. As a consequence of the steep roll-off (and non-linear phase response), we see oscillations in the temporal response which are the most significant for the $H_n$ = 105 µm structure. Thus, if the CPS-SSPP structures are to be used as low-pass filters, then it is important to consider the applications’ phase response requirements.

The results presented in Fig. 6 validate the CPS-SSPP structure proposed in^15^ at THz frequencies. Also, to the authors’ knowledge, this work presents the first demonstration of the SSPP phenomenon using a thin Si$_3$N$_4$ substrate which enables measurements at THz frequencies. There are many opportunities for optimizing CPS-SSPP devices for novel sensing, filtering, and waveguiding applications which we hope to explore in the future.

## Conclusion

This paper presented the experimental verification of several CPS-SSPP structures at THz frequencies using our TSoC platform. Specifically, we fabricated three CPS-SSPP devices on a thin Si$_3$N$_4$ membrane and observed agreement of the band-edge frequencies when $H_n$ = 45 µm (1.04 THz), $H_n$ = 85 µm (0.63 THz), and $H_n$ = 105 µm (0.53 THz). This experimental verification will enable others to investigate a multitude of other novel guided-wave SSPP structures for a range of applications at THz frequencies. As previously noted, the majority of SSPP literature focuses on simulation or GHz scaling for verification. At GHz frequencies, the ability to investigate the vibrational resonances of molecules is limited, and thus a primary sensing application (i.e. THz spectroscopy) is not viable. The methods used in this paper require no device (or frequency) scaling and thus provide a clear path toward integrated SSPP sensors.

Regarding novelty, to the authors’ knowledge, this work demonstrates the first experimental results that confirm SSPP behavior beyond 1 THz using guided wave feedlines for excitation. This capability is possible because the TSoC platform uses an ultra-thin (1 µm) Si$_3$N$_4$ substrate to significantly reduce radiation losses and dispersion (novel for SSPP devices). Lastly, this is the first work to experimentally characterize several different band-edge frequencies within the THz gap which enables the validation of simulation and theoretical models.

## Methods

### Fabrication

The fabrication of the SSPP structures was performed on a Silicon wafer with 1 µm Silicon Nitride coated layer and selectively etched the Silicon from the structure areas to make the thin membrane as illustrated in Fig. 1.

The fabrication of photo-conductive switches (PCSs) and the measurement process are presented in this section. PCSs are used for THz signal generation and detection. These components were fabricated using a multi-step process involving photolithography, gold (Au) sputtering, and wet-etching. The process is detailed in other works^32^, but we summarize it here for completeness. The desired LT-GaAs layer is grown on a sacrificial AlAs layer on a semi-insulating GaAs substrate. The LT-GaAs surface is patterned with Au contacts using standard photolithography. Afterward, each PCS region is masked and wet-etched using Citric Acid and Hydrogen Peroxide (this defines the PCS thickness). Next, the surface is cleaned and re-masked with an etch-resist wax and then submerged in hydrofluoric acid (HF) to detach the LT-GaAs layer by dissolving the AlAs layer. Next, the LT-GaAs film is re-submerged in Citric Acid and Hydrogen Peroxide to disconnect the remaining LT-GaAs film which interconnects a grid of PCSs. Lastly, the etch-resistant wax is removed using trichloroethylene (TCE), resulting in thousands of LT-GaAs PCS active regions. Subsequently, two PCS active regions (transmitter and receiver) were bonded to the TSoC using the Van der Waals (VDW) technique, as explained in^33^. The dimensions of the fabricated PCS are 70 µm $\times$ 40 µm $\times$ 1.8 µm, with 5 µm gap between the metal contacts. The placement of the PCS devices on the structure was performed using a modified probe station. Next, a droplet of water was placed on the PCSs to make robust contact with Van-der-Waals forces. Subsequently, the wafer was mounted on the modified THz-TDS setup to measure the dark current at the ports and then aligned the device position to focus the laser beam on the PCS and achieve the maximum photo-current. Afterward, the THz pulse traveled through the waveguide, and the received signal was measured using a lock-in amplifier. We estimate the optical-to-THz average power conversion efficiency based on the study in^34^. The conversion efficiency of the transmitter PCS considering 24 V bias voltage, 40 µm width of the device, and 7 mW of optical power to be approximately 0.006% which corresponds to a maximum average THz power of 0.42 µW delivered to the CPS TL and subsequent CPS-SSPP.

### Measurement

**Figure 8:**
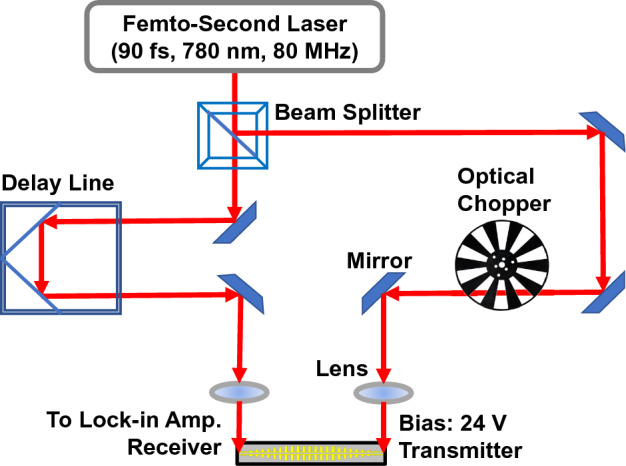
The modified THz time domain spectroscopy setup for performing measurements on THz circuits with photo-conductive switches.

After fabrication of the PCS devices and the CPS-SSPP structures, measurements were performed using the modified THz Time-domain Spectroscopy (THz-TDS) setup depicted in Fig. 8. The experiment involved utilizing a femtosecond pulsed laser with a wavelength of 780 nm focused on PCSs placed on the waveguide to generate and detect a broadband THz pulse signal. The transmitted signal was reconstructed by translating the mechanical delay line measuring the receiver current using a lock-in amplifier (alike THz-TDS)^26,35^. The fluctuations in the measurement are the result of uncorrelated system noise sources (laser, electric, environmental, etc.). A straightforward method to reduce these fluctuations is to perform spectral averaging by measuring each device many times. We note that we did not perform any spectral averaging in this work.

### Supplementary Information

Supplementary Information 1.

Supplementary Information 2.

## Data Availability

All data generated or analyzed during this study are included in this published article and its supplementary information files.
