# Supplementary material for: Demonstration of a terahertz coplanar-strip spoof-surface-plasmon-polariton low-pass filter
Source: Sci Rep. 2024 Jan 2;14:182. doi: 10.1038/s41598-023-50599-y (PMC10762113; doi:10.1038/s41598-023-50599-y)
Supplement: Supplementary file 2 — Supplementary Information 2. [file 41598_2023_50599_MOESM2_ESM.pdf]

Supplementary Material for the manuscript: Demonstration of a Terahertz Coplanar-Strip Spoof-Surface-Plasmon Polariton Low Pass Filter

Mohsen Haghighat[1,2], Thomas Darcie[1,2], Levi Smith[1,2,*]

[1]University of Victoria, Department of Electrical and Computer Engineering, Victoria, BC, V8P 5C2 Canada
[2]Centre for Advanced Materials and Related Technology (CAMTEC) at the University of Victoria, 3800 Finnerty Rd, Victoria, BC, V8P 5C2, Canada.
[*]levismith@uvic.ca

S1 - Impact of Transition Circuit

Fig. 1 plots the simulated signal transmission for CPS-SSPP structure with and without a TC. When a TC is used the stop-band attenuation is significant which occurs when the SSPP mode is excited. Alternatively, when a TC is negated, the stop-band attenuation is less pronounced indicating poor mode conversion. Optimization of the TC is a novel research direction, but it is outside the scope of this paper.

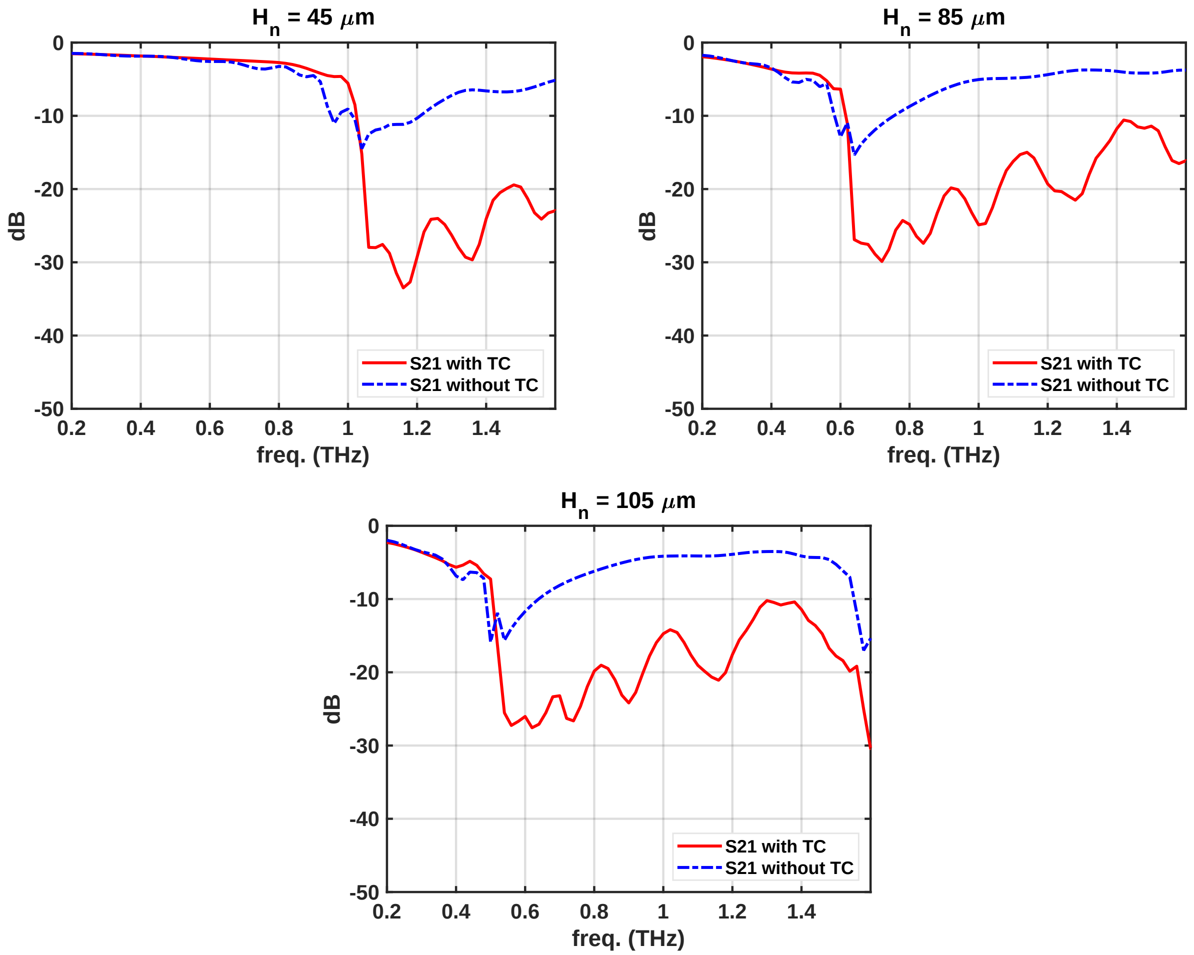

Figure 1. Effect of the transition circuit (TC) on the frequency response.

S2 - Impact of Substrate

We investigate the impact of using another substrate for comparison and to demonstrate the impact on the band-edge frequency. In[1], the authors use a 20 µm thick Polyimide substrate opposed to our 1 µm thick Si_3N_4 substrate. We simulate both substrates using identical conductor configurations and observe the impact of the substrate difference. Figure 2 plots the result where a frequency shift of ≈0.15 THz is observed which occurs due to the different propagation constants.

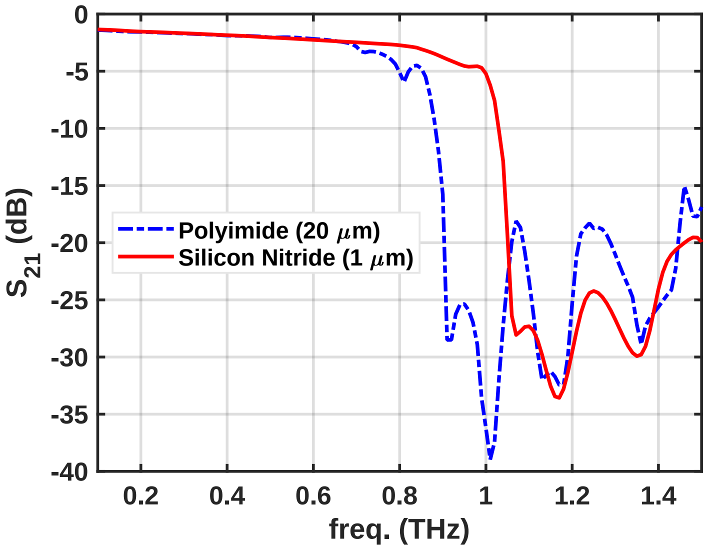

Figure 2. Frequency Response of the CPS-SSPP with H_n = 45 µm on different substrates, Silicon Nitride (red line) and Polyimide (dashed blue line).

References

1. Guo, Y. J., Da Xu, K. & Tang, X. Spoof plasmonic waveguide developed from coplanar stripline for strongly confined terahertz propagation and its application in microwave filters. *Opt. Express* **26**, 10589 (2018).
